# Comparative analysis of the expansion rate and soil erodibility factor of some gullies in Nnewi and Nnobi, Southeastern Nigeria

**DOI:** 10.1038/s41598-023-42320-w

**Published:** 2023-09-15

**Authors:** Stella Kosi Nzereogu, Ogbonnaya Igwe, Chukwuebuka Odinaka Emeh, Kelechi Paulinus Ukor, Pearl Elochukwu Echezona

**Affiliations:** 1 Department of Geology, University of Nigeria, Nsukka, Enugu State Nigeria; 2 Department of Geological Sciences, Nnamdi Azikwe University, Awka, Anambra State Nigeria

**Keywords:** Natural hazards, Solid Earth sciences

## Abstract

The research focused on assessing the expansion rate and soil erodibility factor (K) of specific gullies located in Nnewi and Nnobi, Southeastern Nigeria. Fifteen representative gullies were studied extensively. The Grain size distribution analysis revealed that the soils are composed of gravel (5.77–17.67% and 7.01–13.65%), sand (79.90–91.01% and 82.47–88.67%), and fines (2.36–4.05% and 3.78–5.02%) for Nnewi and Nnobi respectively. The cohesion and internal friction angle values range from 1–5 to 2–5 kPa and from 29–38° to 30–34° for Nnewi and Nnobi respectively, which suggests that the soils have low shear strength and are susceptible to shear failure. The plasticity index (PI) of the fines showed that they are nonplastic to low plastic soils and highly liquefiable with values ranging from 0–10 to 0–9% for Nnewi and Nnobi respectively. Slope stability analysis gave factor of safety (FoS) values in the range of 0.50–0.76 and 0.82–0.95 for saturated condition and 0.73–0.98 and 0.87–1.04 for unsaturated condition for both Nnewi and Nnobi respectively indicating that the slopes are generally unstable to critically stable. The erosion expansion rate analysis for a fifteen-year period (2005–2020) revealed an average longitudinal expansion rate of 36.05 m/yr and 10.76 m/yr for Nnewi and Nnobi gullies respectively. The soil erodibility factor (K) are 8.57 × 10^−2^ and 1.62 × 10^−4^ for Nnewi and Nnobi respectively indicating that the soils in Nnewi have higher erodibility potentials than those of Nnobi. Conclusively, the Nnewi area is more prone to erosion than the Nnobi area.

## Introduction

**Figure 1:**
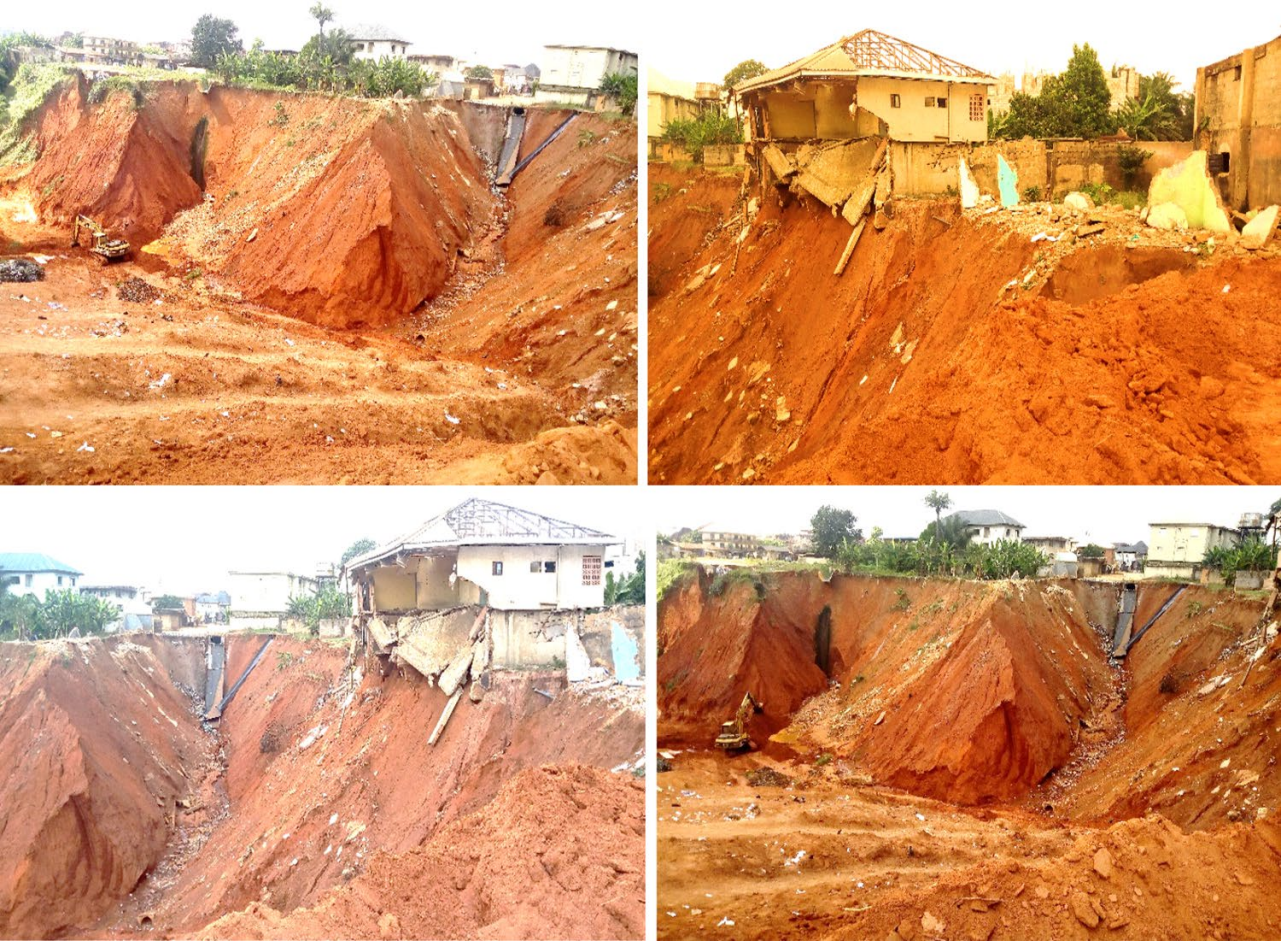
A gully erosion site in the study area.

In southeastern Nigeria, the persistent threat of natural environmental hazards, including landslides and soil erosion, jeopardizes both lives and property within the region^1–3^. Within this context, the regions of Nnewi and Nnobi in Anambra state have fallen prey to the deleterious effects of large gully erosions, emerging as a pronounced menace to local residents (Fig. 1). Gully erosion, a process driven by swiftly flowing water eroding soil and forming channels, becomes increasingly concerning during the wet season. The heightened slope instability during this time often leads to the failure of steep gully slope walls due to rising pore pressure levels^4–6^.

The yearly peak of the rainy season serves as a critical point, amplifying the impact of gully erosion as it widens its catchment area^5–7^. Against this backdrop, the need to examine the soil loss rate and the potential consequences of urbanization on gully erosion’s magnitude and intensity becomes more pressing in Nnewi and Nnobi^7–9^. This study reveals the underlying causes of the 2019 building collapse attributed to gully erosion in Okpunor-Egbu Umudim, Nnewi and unravels the reasons behind the differing levels of gully erosions between Nnewi, a densely populated town, and its less populated neighbour, Nnobi, both located within Anambra State.

The distinction in the magnitude and intensity of gully erosions between Nnewi and Nnobi has not gone unnoticed^4,5,10–13^. This observation prompted a more comprehensive investigation to discern the factors responsible for these disparities, considering their comparable geomorphological and climatic attributes. This inquiry raises pertinent questions: (i) What drives the observed variation? (ii) Which factors contribute to the higher soil loss rate in Nnewi? (iii) How can these factors be precisely identified?

These queries lead to the formulation of a hypothesis that attributes the observed variations to the accelerated urbanization and population growth in Nnewi^4,14–16^ compared to the less densely populated Nnobi. Urban expansion often involves deforestation for commercial and residential purposes, disrupting natural drainage patterns and exacerbating surface runoff in highly populated areas like Nnewi^14,15^. In contrast, the presence of trees in Nnobi mitigates the force of surface runoff, which is the primary catalyst for gully erosion^4,14^. The roots of these trees bind the soil particles, reducing the destructive impact of surface runoff during the rainy season^4^.

Gully erosion, triggered by fast-flowing water eroding soil and creating channels, is exacerbated by increased slope instability during wet seasons, causing steep gully slope walls to fail due to heightened pore pressure levels^4,5,12,13^. The vulnerability of soil to water erosion is determined by geological, geomorphological, and climatic conditions within the area^4,13^.

Researchers such as Igwe and Fukuoka and Igwe et al. confirm that soil saturation during peak wet seasons increases pore pressure, reducing soil shear strength and rendering it susceptible to failure through liquefaction^17,18^. Additionally, intensified rainfall augments soil erodibility^4^. Egbueri and Igwe established that Eocene Nanka Sands (Imo Basin) and Cretaceous Ajali Sandstone (Anambra Basin) are highly susceptible to erosion due to their unique soil gradation^8^. This susceptibility is further exacerbated by undulating topography, leading to the formation of large, steep gullies^4^. Rainfall amplifies existing gullies as surface runoff erodes more soil^19^. The influx of pollutants from increased population and urbanization contributes to alkaline surface runoff, further eroding soil^12^.

While previous research has explored the occurrence and causes of soil erosion in southeastern Nigeria, none have ventured into comparing the expansion rate of gullies and estimating the soil erodibility factor of soils within Nnewi and Nnobi. This research aims to analyze the expansion rate and soil erodibility factor (K) of select gullies in Nnewi and Nnobi, Southeastern Nigeria. The primary objectives include evaluating the geotechnical properties impacting soil erodibility and gully intensity, calculating the gully expansion rate and soil erodibility factor (K), developing a slope failure model, and determining the factor of safety of the gully slope material.

## The setting of the study area

**Figure 2:**
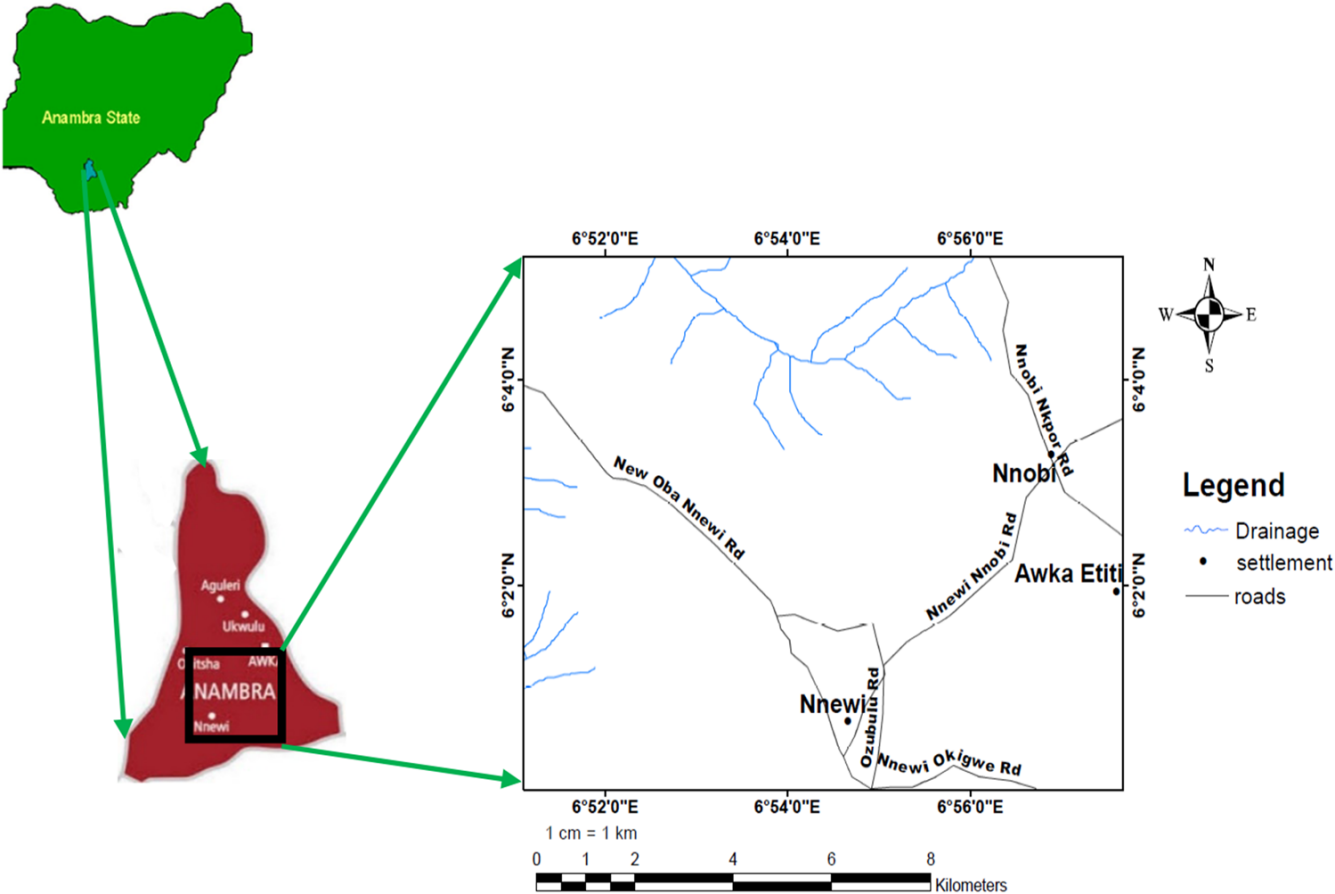
Location and accessibility map of the study area. Created using the ArcGIS and ArcScene software (version 10.4.1).

The study area is located at Nnewi and Nnobi in Anambra State (Fig. 2). It is bound by Latitudes 6°0′ 0ʺ N to 6° 6′ 0ʺ N and Longitudes 6° 50′ 0ʺ E to 6° 58′ 0ʺ E. Major towns in the study area includes Nnewi, Nnobi, and Awka Etiti. The southern part of the study area can be easily accessed through the Nnewi-Okigwe and New Oba-Nnewi roads which run essentially Northwest-Southeast of the area. The Northern part of the study area can be accessed through the Nnobi-Nkpor road.

**Figure 3:**
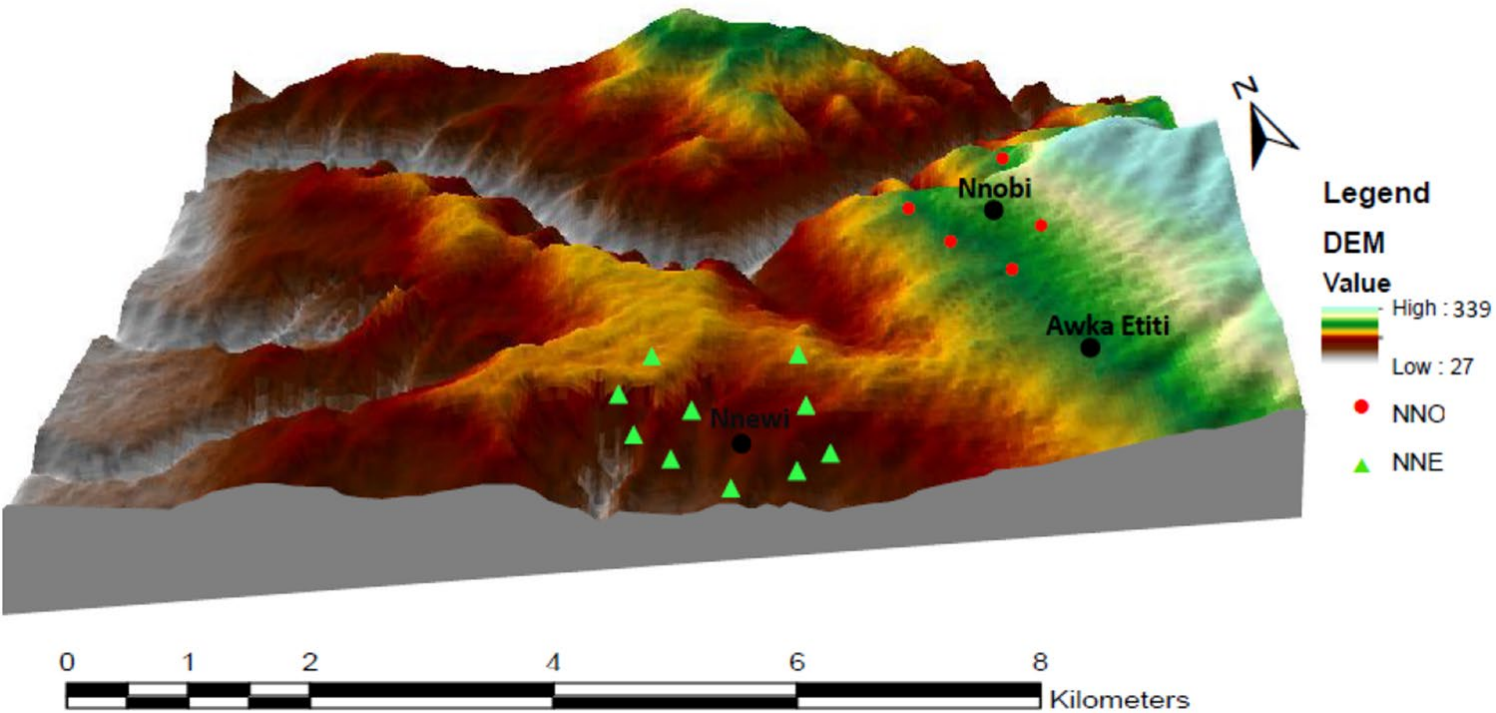
3D DEM of the study area showing drainage channels. Created using the ArcGIS and ArcScene software (version 10.4.1).

The area has an undulating topography comprising of gently inclined hilltops to very steep V-shaped valleys cut by seasonal drainage channels and gullies in the study area (Fig. 3). The dendritic drainage pattern in the area can be associated with the uniformity of the underlying lithology. The inherent weakness of the lithology combined with the down-cutting action of the seasonal drainages have shaped the area into a jagged topography it has today.

The study area is within the humid tropical climate; it is characterized by two seasons: the rainy and dry seasons. The wet season spans from late March or early April till the end of October or early November, while the dry season is usually from November to March. The mean monthly temperatures vary from 22 to 28 °C in the wet season and between 28 and 32 °C in the dry season. The annual rainfall is between 1500 and 2100 mm^4^. The average monthly rainfall for 30 years ranges from less than 1 mm in the dry season to about 300 mm in the rainy season^20^. The wet periods are characterized by moderate temperatures and high relative humidity, while the dry periods have high temperatures and lower relative humidity^20^.

The study area falls in the tropical rainforest belt^21^, and is largely dominated by short grasses at the elevated areas with thick forests of tall trees in the valleys and along drainage channels.

**Figure 4:**
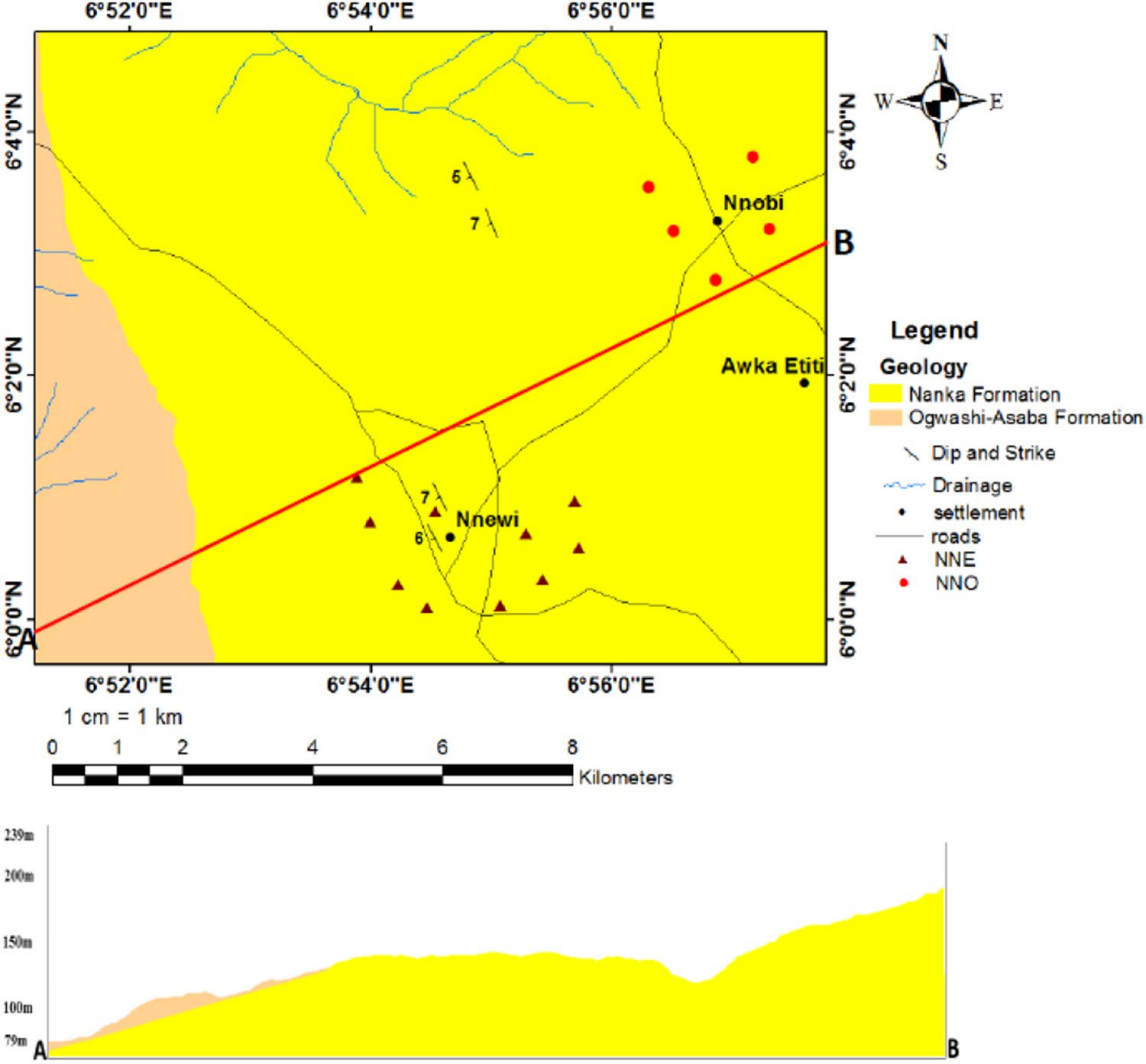
Geologic map of the study area. Created using the ArcGIS and ArcScene software (version 10.4.1).

The study area is underlain by loose Nanka Formation (Fig. 4) which is part of the Ameki Group. Ameki Group comprises three lateral equivalents: Ameki Formation, Eocene Nanka Formation and Nsugbe Sandstone^22^. The Nanka Formation is predominantly composed of loose and poorly consolidated fine-grained sand materials with low clay content and little or no coarse-grained aggregates^21^. It is noted as the loose sand facies of the Ameki Group^22^, outcrops over an area in excess of 1400 km exclusively east of the Niger and noted to be very susceptible to erosion. The Formation is made up of loose, tabular to planar cross bedded, flaser-bedded, fine to medium grained sand, with a few mudrock breaks. The sand consists of sub-rounded to sub-angular grains and has an average of 5% clay content, which makes it texturally submature. It is however compositionally mature because of the absence of feldspars^22^.

## Materials and methods

The materials used in this work were soils obtained from gully sites around Nnewi and Nnobi towns both in Anambra State. A total of 30 gully erosion sites were visited in both towns and 15 representative soil samples were collected: 10 soil samples from Nnewi and 5 soil samples from Nnobi. The soil samples were put in well labelled sampling bags and sealed to minimize the loss of natural soil moisture through evaporation.

The detailed field study was carried out in February 2021. It involved detailed geologic descriptions of outcropping units with emphases on their texture, colour and structures.

At every sampling point, about 5 kg of soil samples were collected after the removal of the top soil from a depth of 25–30 cm to ensure that a true and fresh representative soil sample devoid of humus and/or artificial fertilizer was obtained. These were carefully put in well-labelled sampling bags to avoid mixing up the location details. A total of fifteen (15) representative samples-ten (10) samples from Nnewi and five (5) from Nnobi were analyzed in the Civil Engineering Laboratory of the University of Nigeria Nsukka. The laboratory analysis to obtain the geotechnical and other properties of the samples such as specific gravity (ASTM D854-14), permeability (ASTM D5084-16), shear strength parameters (ASTM D3080-11)- cohesion (C) and angle of internal friction (Ø), particle size distribution, Optimum Moisture Content (OMC) using ASTM D698-12, Maximum Dry Density (MDD) using ASTM D698-12, Natural Moisture Content (NMC) using ASTM D2216-19, Atterberg limits using ASTM D4318-17-Liquid Limit (LL), Plastic Limit (PL) and Plasticity Index (PI) were determined. All were carried out in accordance with American Society for Testing and Materials (ASTM) standards for soil. The results obtained from the field and laboratory were integrated into Slope/W Geostudio 2012 software to produce a modeled diagram of the gully slope morphology and failure prediction on the slope. The integrated results are the slope height, width and slope angles obtained from the field and shear strength parameters (cohesion and angle of internal friction), unit weight obtained from the laboratory. The Morgenstern-Price limit equilibrium method was used to run the slope stability analysis. Aerial photographs from Google Earth Pro were used to monitor the erosion sites (gullies) for a fifteen year period for both Nnewi and Nnobi areas. The gully expansion rates (longitudinal and width) were determined using dimensions obtained with the ruler tool on Google Earth Pro.

### Calculation of soil erodibility factor (K)

The soil erodibility factor (K) was determined using the formula introduced by Jain and Singh^24^. This factor characterizes a soil’s susceptibility to erosion and is integral to erosion prediction models such as the Revised Universal Soil Loss Equation (RUSLE). The calculation involved the following steps:

1. *Parameters and equations*: The soil erodibility factor (K) is expressed as:

$$ {\text{K}}_{{{\text{fact}}}} = 1.292 \, [2.1 \times 10^{ - 6} {\text{F}}_{{\text{p}}}^{1.14} \left( {12 - {\text{P}}_{{{\text{om}}}} } \right) \, + \, 0.0325 \, \left( {{\text{S}}_{{{\text{struc}}}} - 2} \right) \, + \, 0.025 \, \left( {{\text{F}}_{{{\text{perm}}}} - 3} \right)] $$

where F_p_ is the particle size parameter, computed as F_p_ = P_silt_ (100—P_clay_). P_om_ is the percentage of organic matter. S_struc_ is the soil structure index. F_perm_ is the profile-permeability class factor. P_clay_ is the percentage of clay content.

2. *Soil Structure and Permeability Class*: Values were assigned to soil structure and permeability class factors based on specific classifications. For soil structure, values were designated as follows:

- 1 = Very fine granular soil
- 2 = Fine granular soil
- 3 = Medium or coarse granular soil
- 4 = Blocky, platy, or massive soil

Similarly, for permeability class, values were assigned as follows:

1 = Very slow infiltration

2 = Slow infiltration

3 = Slow to moderate infiltration

4 = Moderate infiltration

5 = Moderate to rapid infiltration

6 = Rapid infiltration

## Results and discussion

### Field observation of gully characteristics

**Figure 5:**
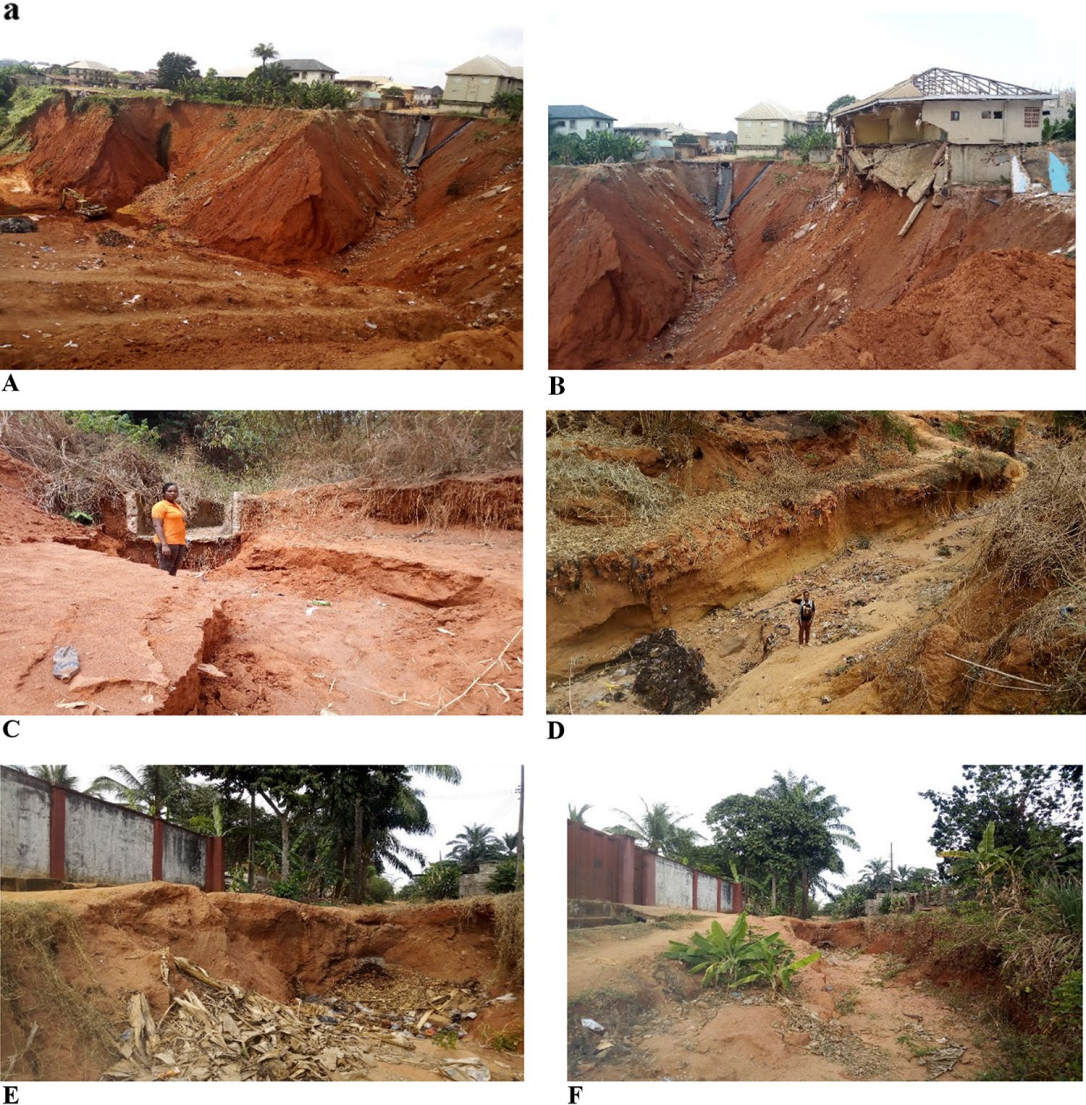

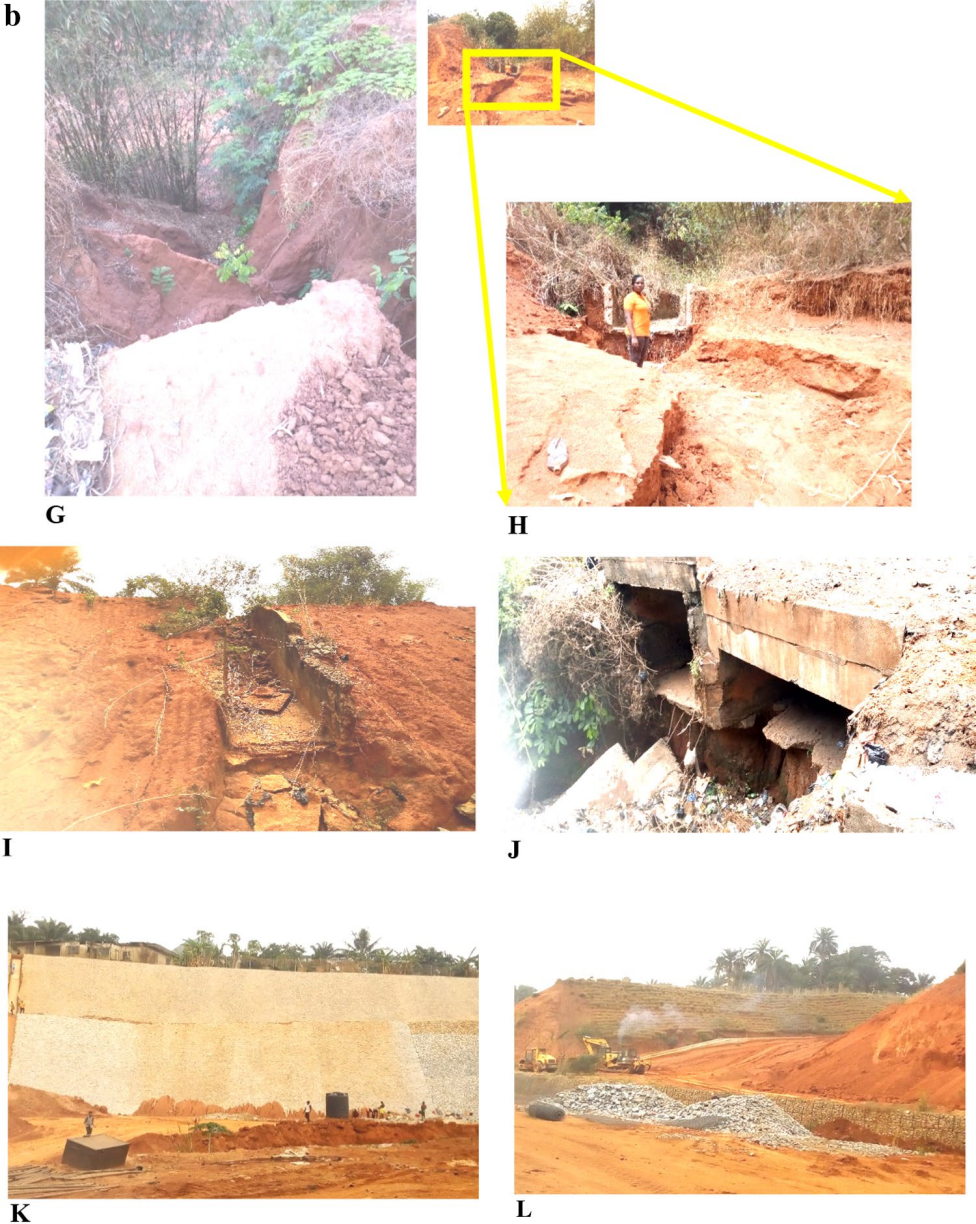
(**a**) Some of the Gullies in the study area A-C) Nnewi D-F) Nnobi. (**b**) Some of the gullies in Nnewi area (G-J) and some mitigating measures (I-J).

The field observations of gully properties are shown in Table 1. The gullies in Nnewi were active, wider and deeper than those in Nnobi, which were mostly dormant and overtaken by tall trees and open dumps for solid wastes (Fig. 5a). The use of vetiver plants (grass) and retaining walls for soil stabilization was observed in some Nnewi locations (NNE1, NNE2, and NNE4) (Fig. 5b). Some of the houses, drainage channels and culvert were observed to have been damaged by the erosive actions of surface runoff. The soils in the study area were clean, loose, fine-medium grained lateritic sands. The little or no fines in them increases their susceptibility to erosion^21^. The gullies in the study area generally trend NW–SE with slope angles ranging from 40° to 78° for Nnewi and 45°–69° for Nnobi area respectively. The relatively higher slope angles measured in Nnewi makes the gully walls more unstable than those in Nnobi. This increases the intensity and rate at which erosion occurs in Nnewi. The steeper slopes help to reduce the soils shear strength making them susceptible to shear failure^23^. Furthermore, Nnobi lies in a relatively higher topography with gentler slopes than Nnewi. This makes surface runoffs more erosive in Nnewi area since water moves from higher elevation to lower elevation^14^.

**Table 1:** Gully slope locations and field observations.

Sample ID	Latitude (N)	Longitude (E)	Elevation (m)	Gully depth (m)	Gully width (m)	Average lateral extent (m)	Slope angle (o)	Lithology
NNE1	06° 3ʹ 16.6ʺ	06° 54ʹ 54.3ʺ	119	39	85	480	65	Loose lateritic sand
NNE2	06° 3ʹ 14.2ʺ	06° 54ʹ 57.8ʺ	116	22	50	80	72	Loose lateritic sand
NNE3	06° 3ʹ 21.9ʺ	06° 54ʹ 54.9ʺ	110	30	79	120	60	Medium-grained sand
NNE4	06° 3ʹ 36.5ʺ	06° 54ʹ 48.4ʺ	72	34	34	62	40	Loose fine-grained sand
NNE5	06° 3ʹ 39.5ʺ	06° 54ʹ 46.5ʺ	62	15	30	112	48	Fine-medium-grained sand
NNE6	06° 0ʹ 38.9ʺ	06° 54ʹ 29.9ʺ	101	65	10	72	64	Loose lateritic sand
NNE7	06° 0ʹ 59.2ʺ	06° 54ʹ 33.2ʺ	101	46	18	123	73	Brownish fine sand
NNE8	06° 1ʹ 2.7ʺ	06° 54ʹ 33.1ʺ	117	10	20	97	70	Loose lateritic sand
NNE9	06° 0ʹ 7.5ʺ	06° 55ʹ 4.5ʺ	100	28	5	150	65	Fine-grained lateritic sand
NNE10	06° 1ʹ 6.6ʺ	06° 54ʹ 47.1ʺ	140	79	60	250	78	Loose lateritic sand
NNO1	06° 3ʹ 42.2ʺ	06° 55ʹ 10.1ʺ	101	12	6	43	45	Fine-grained lateritic sand
NNO2	06° 3ʹ 42.0ʺ	06° 55ʹ 12.3ʺ	134	14	3	20	54	Loose lateritic sand
NNO3	06° 3ʹ 40.5ʺ	06° 55ʹ 23.8ʺ	146	25	10	50	69	Loose fine-grained sand
NNO4	06° 2ʹ 35.8ʺ	06° 56ʹ 35.1ʺ	177	13	4	28	51	Loose lateritic sand
NNO5	06° 2ʹ 32.1ʺ	06° 56ʹ 24.8ʺ	175	8	4	10	46	Brownish-yellow sand

### Particle size distribution (grain size analysis)

**Figure 6:**
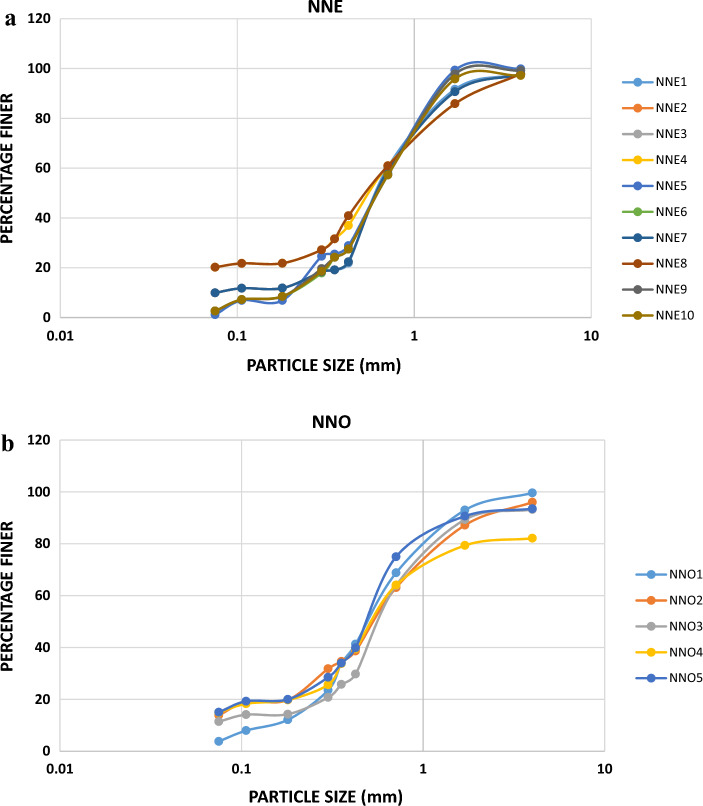
(**a**) Particle size distribution curve for Nnewi area. (**b**) Particle size distribution curve for Nnobi area.

The particle size distribution results are presented in Table 2. The results revealed that the soils are composed of gravel (5.77–17.67%), sand (79.90–91.01%), and fines (2.36–4.05%) for Nnewi area and gravel (7.01–13.65%), sand (82.47–88.67%), and fines (3.78–5.02%) for Nnobi area. The soils in the study area generally have high sand percentage and low percentage of fines and gravel. The dominance of sand and low fine and gravel content of the soils (Fig. 6a,b) make them very susceptible to erosion since the cohesion among sand grain is little^5,14,21^. The relative higher fine percentage in Nnobi may also be the reason for less gully intensity since the cohesive forces between grains (supplied by the fine content) are higher.

**Table 2:** Particle size distribution and soil classification of the study area.

Sample ID	Gravel (%)	Sand (%)	Fines (%)	Cc	Cu	USCS	Sample ID	Gravel (%)	Sand (%)	Fines (%)	Cc	Cu	USCS
NNE1	8.94	87.95	3.11	1.04	2.89	SP	NNO1	8.54	87.68	3.78	1.14	3.65	SP
NNE2	12.34	83.61	4.05	1.11	1.32	SC	NNO2	13.65	82.49	3.86	1.06	3.15	SP
NNE3	17.67	79.90	2.43	1.09	2.57	SP	NNO3	7.01	87.97	5.02	1.98	4.02	SC
NNE4	10.04	86.65	3.31	1.02	1.43	SP	NNO4	10.51	85.56	3.93	1.13	3.33	SW
NNE5	10.46	86.48	3.06	0.98	1.95	SP	NNO5	7.32	88.67	4.01	1.12	3.97	SC
NNE6	5.77	91.01	3.22	1.23	1.45	SP							
NNE7	8.98	87.78	3.24	1.17	3.78	SW							
NNE8	13.87	83.77	2.36	1.13	2.32	SP							
NNE9	12.97	83.92	3.11	1.22	1.92	SP							
NNE10	10.15	87.18	2.67	1.09	1.03	SP

In addition, the values of the coefficient of uniformity (Cu) and coefficient of curvature (Cc) ranged from 1.03–3.78 to 0.98–1.23 for Nnewi and 3.15–4.02 and 1.06–1.98 for Nnobi respectively (Tables 2, 3) indicating that the soils are poorly graded. According to Arora, well-graded soils have Cu > 6 and Cc ranging from 1 to 3^23^. Since the shear strength of well-graded soils are higher^23^, it follows that the soils in the study area have low shear strength.

**Table 3:** Summary statistics of particle size distribution analysis.

Sample ID (NNE1-10)	Gravel (%)	Sand (%)	Fines (%)	Cc	Cu	Sample ID (NNO1-5)	Gravel (%)	Sand (%)	Fines (%)	Cc	Cu
MAX	17.67	91.01	4.05	1.23	3.78	MAX	13.65	88.67	5.02	1.98	4.02
MIN	5.77	79.9	2.36	0.98	1.03	MIN	7.01	82.49	3.78	1.06	3.15
MEAN	11.12	85.83	3.06	1.11	2.07	MEAN	9.41	86.47	4.12	1.29	3.62

Based on the Unified Soil Classification Scheme (USCS) as shown in Table 2, the soil types in the study area vary from poorly graded sand (SP), clayey sand (SC) and well-graded sand (SW). However, the most dominant type is poorly graded sand, which makes the area prone to shear failure because of its low shear resistance^23^.

### Specific gravity

The fifteen soil samples in the study area are predominantly sands with little gravel and fines, indicating that they possess similar range of specific gravity values. The specific gravity results (Table 4) for Nnewi and Nnobi ranged from 2.45–2.66 to 2.54–2.78 respectively. This indicate the soils have similar erodibility properties^5,21^.

**Table 4:** Specific gravity of the soil samples in the study area.

Sample ID	Specific gravity	Sample ID	Specific gravity
NNE1	2.52	NNO1	2.78
NNE2	2.51	NNO2	2.65
NNE3	2.47	NNO3	2.54
NNE4	2.46	NNO4	2.67
NNE5	2.59	NNO5	2.55
NNE6	2.52		
NNE7	2.45		
NNE8	2.56		
NNE9	2.66		
NNE10	2.48		

### Coefficient of permeability

The permeability (k) of a soil is a direct indication of the infiltration capacity of that soil^23^. The higher the permeability of the soil, the higher the infiltration capacity and ease at which they can be washed away by running water. The soils in the study area have permeability values ranging from 2.92 × 10^−5^ to 6.80 × 10^−4^ m/s and 2.35 × 10^−6^ to 3.84 × 10^−4^ m/s for Nnewi and Nnobi respectively (Table 5). According to Arora, soils with permeability values falling within the range of 10^−7^ to 10^−5^ m/s are categorized as moderately to highly permeable^23^. This shows that the soils in the study are moderately to highly permeable. The high permeability and the predominance of sands in the study area makes it very vulnerable to erosion especially at the peak of the wet season when the rainfall intensity is maximum. Furthermore, the relatively lower permeability observed in Nnobi samples (NNO2 and NNO3) was because of their higher fine content making them more resistant to erosion than Nnewi area. As rainfall infiltrates into these highly permeable soils, the pore pressure increases thereby reducing the shear strength of the material^23^. This leads to shear failure and increased rate of instability in the study area.

**Table 5:** Permeability (m/s) of the soil samples in the study area.

Sample ID	Permeability (m/s)	Sample ID	Permeability (m/s)
NNE1	6.80 × 10^−4^	NNO1	3.84 × 10^−4^
NNE2	2.92 × 10^−5^	NNO2	3.07 × 10^−5^
NNE3	2.77 × 10^−4^	NNO3	2.35 × 10^−6^
NNE4	4.80 × 10^−5^	NNO4	2.38 × 10^−5^
NNE5	5.98 × 10^−4^	NNO5	2.79 × 10^−5^
NNE6	3.59 × 10^−4^		
NNE7	4.47 × 10^−5^		
NNE8	2.47 × 10^−4^		
NNE9	6.72 × 10^−4^		
NNE10	5.30 × 10^−5^		

### Compaction characteristics of the soils in the study area

**Figure 7:**
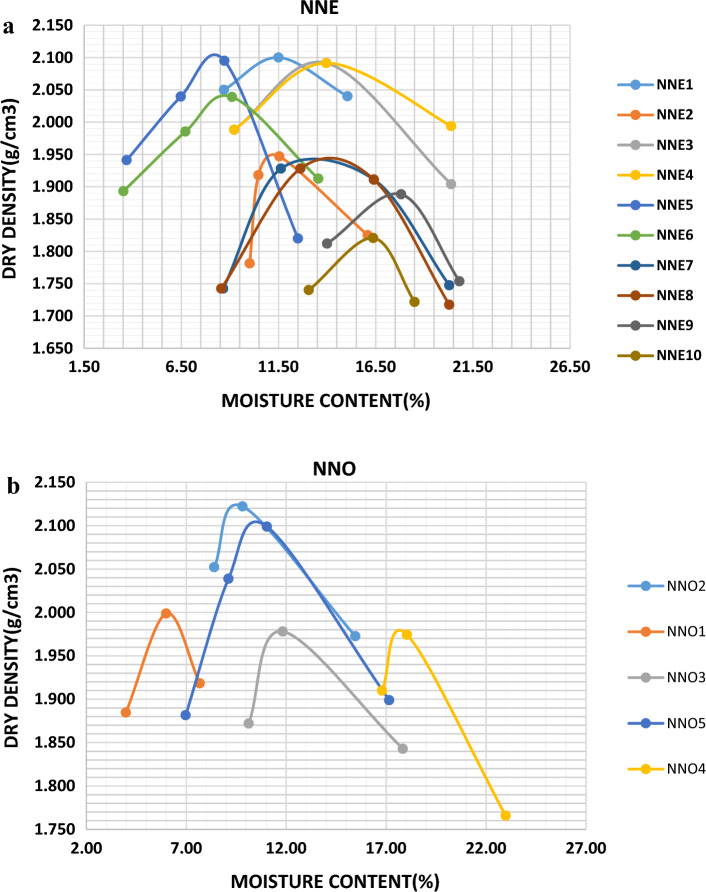
(**a**) Compaction curves of the soil samples in Nnewi area. (**b**) Compaction curves of the soil samples in Nnobi area.

Compaction test is done to reveal how loose or dense (compacted) a soil sample is. The denser the soil, the higher the internal friction angle between the grains and the higher the shear strength of the material^19,23^. The results of the compaction test are presented in Table 6 with the compaction curves shown in Fig. 7a,b for Nnewi and Nnobi respectively. From the results, the values of maximum dry density (MDD) and optimum moisture content (OMC) ranged from 1.82–2.11 g/cm^3^ and 8.20–17.81% for Nnewi and 1.98–2.13 g/cm^3^ and 6.00–17.80% respectively. The result reveals that the soils have moderate to high OMC and low MDD. This suggests that the soils from both area are loose, lack ability for structural support (bearing capacity) hence, susceptible to erosion. However, soils in Nnewi area with lower dry density are more prone to erosion compared to Nnobi area. This agrees with the findings of Igwe and Egbueri that loose soils require very little force to detach and transport the particle^21^.

**Table 6:** Compaction test result of the soil samples in the study area.

Sample ID	OMC (%)	MDD (g/cm^3^)	NMC (%)	Sample ID	OMC (%)	MDD (g/cm^3^)	NMC (%)
NNE1	11.50	2.10	3.00	NNO1	6.00	2.00	2.00
NNE2	11.20	1.95	3.00	NNO2	9.50	2.13	2.00
NNE3	13.40	2.10	3.00	NNO3	11.50	1.98	4.00
NNE4	13.42	2.11	3.00	NNO4	17.80	1.98	4.00
NNE5	8.20	2.11	2.00	NNO5	10.50	2.11	3.00
NNE6	8.50	2.04	2.00				
NNE7	14.28	1.96	3.00				
NNE8	14.30	1.94	3.00				
NNE9	17.81	1.89	4.00				
NNE10	16.50	1.82	3.00

### Natural moisture content and Atterberg limits

The natural moisture content (NMC) of the soil gives an indication of its shear strength and load bearing capacity^23^. The NMC values of the soils in the study area (Table 6) ranged from 2 to 4% for both Nnewi and Nnobi areas. This shows that the soils have low water saturation and thus, low cohesive attractive forces among the soil grains^26^.

**Figure 8:**
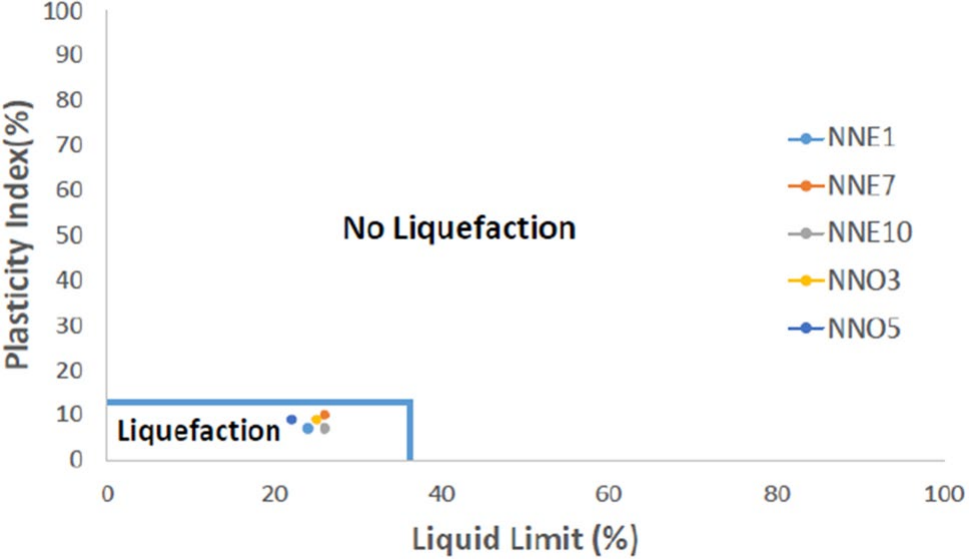
Liquefaction chart of the study area (after Seed et al.^27^).

The results of the Atterberg limits as presented in Table 7 reveals that the liquid limit (LL) ranges from NP-26%, the plastic limit (PL) from NP-19% and the plasticity index (PI) from NP-10% for Nnewi area while for Nnobi area, the LL ranges from NP-25%, PL ranges from NP-16% and PI ranges from NP-9%. Generally, the plasticity index (PI) of the fines showed that they are nonplastic (NP) to low plastic soils and highly liquefiable. According to Seed et al., soils with PI values less than 12% and LL values less than 37% are easily liquefiable (Fig. 8). From the plot, the soils are all susceptible to liquefaction and shear failure^27^.

**Table 7:** Atterberg limit test result.

Sample ID	LL (%)	PL (%)	PI (%)	SAMPLE ID	LL (%)	PL (%)	PI (%)
NNE1	24	17	7	NNO1	25	NP	NP
NNE2	22	NP	NP	NNO2	NP	NP	NP
NNE3	NP	NP	NP	NNO3	25	16	9
NNE4	25	NP	NP	NNO4	NP	NP	NP
NNE5	NP	NP	NP	NNO5	22	13	9
NNE6	24	NP	NP				
NNE7	26	16	10				
NNE8	NP	NP	NP				
NNE9	NP	NP	NP				
NNE10	26	19	7

### Shear strength characteristics of the soils

**Figure 9:**
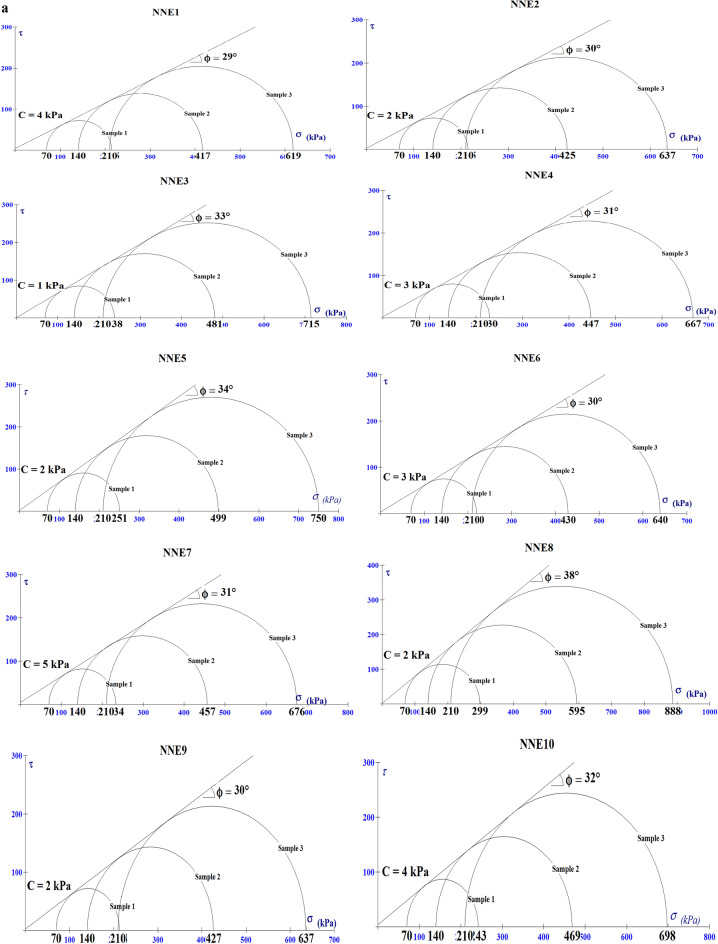

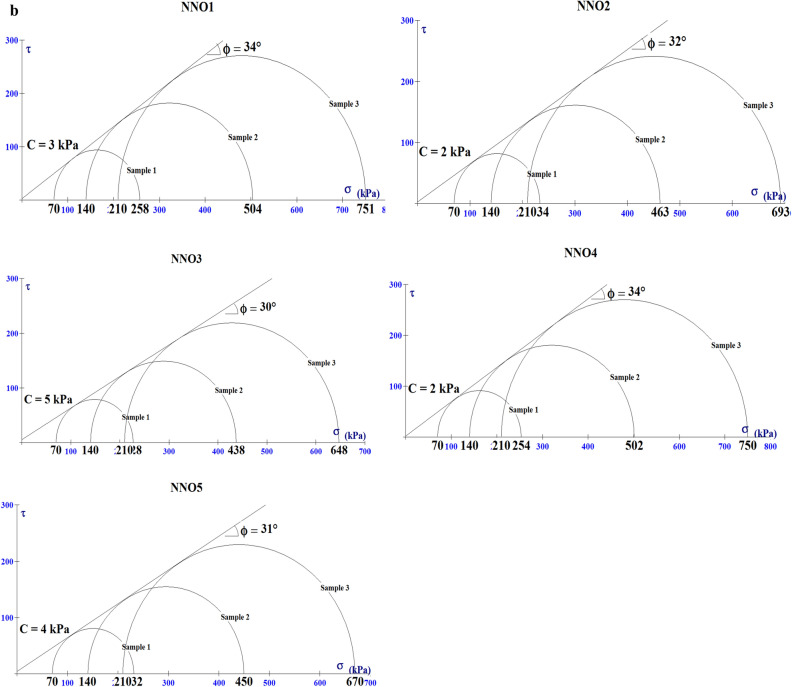
(**a**) Failure envelop of soil samples in Nnewi area. (**b**) Failure envelop of soil samples in Nnobi area.

The cohesion (C) and internal friction angle (Ø) are very important parameters in the determination of the shear strength of materials. According to Arora, the higher the Ø in sands, the higher the degree of interlocking among grains and consequently, the higher the shear strength (ability to withstand shear failure)^23^. Furthermore, the lower the shear strength of the material the higher their erodibility. The values of the shear strength parameters (C and Ø) of the analysed soils are presented in Table 8 and Fig. 9a,b. All the soils have low cohesion values ranging from 1–5 to 2–5 kPa and internal friction angle ranging from 29°–38° to 30°–34° for Nnewi and Nnobi respectively, which suggests that the soils have low shear strength as cohesion is a major component of shear strength equation^23^ and are susceptible to shear failure^14,21,25^.

**Table 8:** The shear strength test results of the soils.

Sample ID	Cohesion (C, Kpa)	Friction angle (Ø,°)	Sample ID	Cohesion (C, Kpa)	Friction angle (Ø,°)
NNE1	4	29	NNO1	3	34
NNE2	2	30	NNO2	2	32
NNE3	1	33	NNO3	5	30
NNE4	3	31	NNO4	2	34
NNE5	2	34	NNO5	4	31
NNE6	3	30			
NNE7	5	31			
NNE8	2	38			
NNE9	2	30			
NNE10	4	32

### Gully slope stability analysis

**Figure 10:**
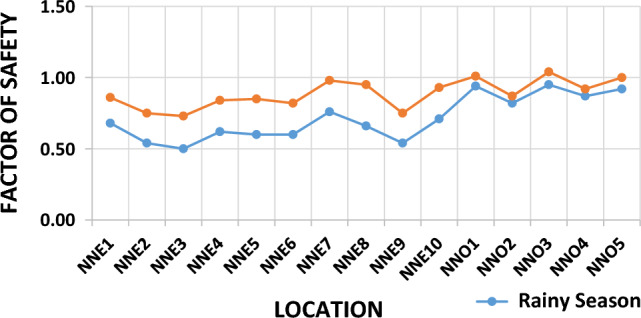
Factor of safety plot of the study area.

**Figure 11:**
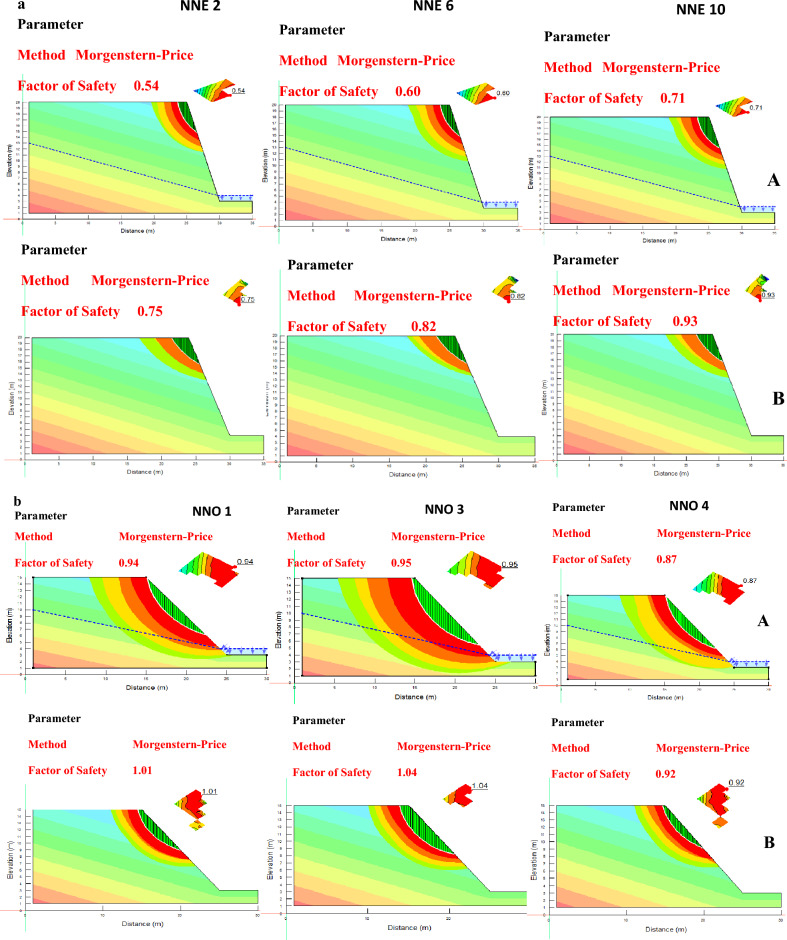
(**a**) Slope stability models of representative slopes in Nnewi (A) wet season (B) dry season. (**b**) Slope stability models of representative slopes in Nnobi (A) wet season (B) dry season.

The stability analysis of the fifteen gully slopes within the study area was conducted to determine their factor of safety (FoS). The FoS values fell within the range of 0.50–0.76 and 0.82–0.95 for the saturated condition, and 0.73–0.98 and 0.87–1.04 for the unsaturated condition, for Nnewi and Nnobi respectively (Fig. 10). The behaviour of these gully slopes during the dry season was represented by the unsaturated condition, while the saturated condition represented their behaviour during the rainy season. The results of the stability analysis for both the saturated (wet season) and unsaturated (dry season) conditions were documented in Table 9. Specifically, the gully slopes labelled as NNE2, NNE6, and NNE10 corresponded to the Nnewi location (Fig. 11a), while gullies NNO1, NNO3, and NNO4 represented the Nnobi location (Fig. 11b). From the analysis, it was revealed that all the gully slopes in Nnewi were determined to be unstable^28^ presented in Table 10. In contrast, in the Nnobi area, 60% of the gullies were found to be unstable, while the remaining 40% were classified as critically stable. To provide a more specific breakdown, the locations NNO1 and NNO3 displayed FoS values of 0.94 and 0.95 during the rainy season, and 1.01 and 1.04 during the dry season, respectively. These values indicated unstable slopes for NNO1 and critically stable slopes for NNO3. This highlights the heightened hazard of the gullies during the rainy season, when the slopes are either partially or fully saturated, aligning with the findings of Igwe and Chukwu^29^.

**Table 9:** Slope stability analysis result.

Location	FoS (dry season)	Remark	Hazard level	FoS (wet season)	Remark	Hazard level
NNE1	0.86	Unstable	High	0.68	Unstable	High
NNE2	0.75	Unstable	High	0.54	Unstable	High
NNE3	0.73	Unstable	High	0.50	Unstable	High
NNE4	0.84	Unstable	High	0.62	Unstable	High
NNE5	0.85	Unstable	High	0.60	Unstable	High
NNE6	0.82	Unstable	High	0.60	Unstable	High
NNE7	0.98	Unstable	High	0.76	Unstable	High
NNE8	0.95	Unstable	High	0.66	Unstable	High
NNE9	0.75	Unstable	High	0.54	Unstable	High
NNE10	0.93	Unstable	High	0.71	Unstable	High
NNO1	1.01	Critically stable	Medium	0.94	Unstable	High
NNO2	0.87	Unstable	High	0.82	Unstable	High
NNO3	1.04	Critically stable	Medium	0.95	Unstable	High
NNO4	0.92	Unstable	High	0.87	Unstable	High
NNO5	1.00	Critically stable	Medium	0.92	Unstable	High

**Table 10:** Slope stability classification based on FoS (after Hadmoko et al*.* 2010).

No	FoS value	Slope stability	Hazard level
1	FoS > 1.5	Stable slope	Low hazard
2	1.0 ≤ FoS ≤ 1.5	Critical slope	Medium hazard
3	FoS < 1.0	Unstable slope	High hazard

Furthermore, the decrease in FoS values during the rainy season was attributed to the rise in pore pressure and unit weight of the soil, resulting in reduced shear strength of the slope materials. This observation was consistent with previous studies by Emeh and Igwe, Igwe and Egbueri, Arora, and Nebeokike et al.^15,21,23,25^.

Incorporating slope angle, it was noted that the steeper angles of the gullies in Nnewi contributed to the higher instability of their walls. This collectively pointed to the conclusion that the gullies in Nnewi were inherently more unstable than those in Nnobi, both during the rainy and dry seasons. The instability of a slope occurs when the activating shear force surpasses the resisting shear strength of the slope materials^23,30^, invariably leading to slope failure.

### Gully expansion rate analysis

**Figure 12:**
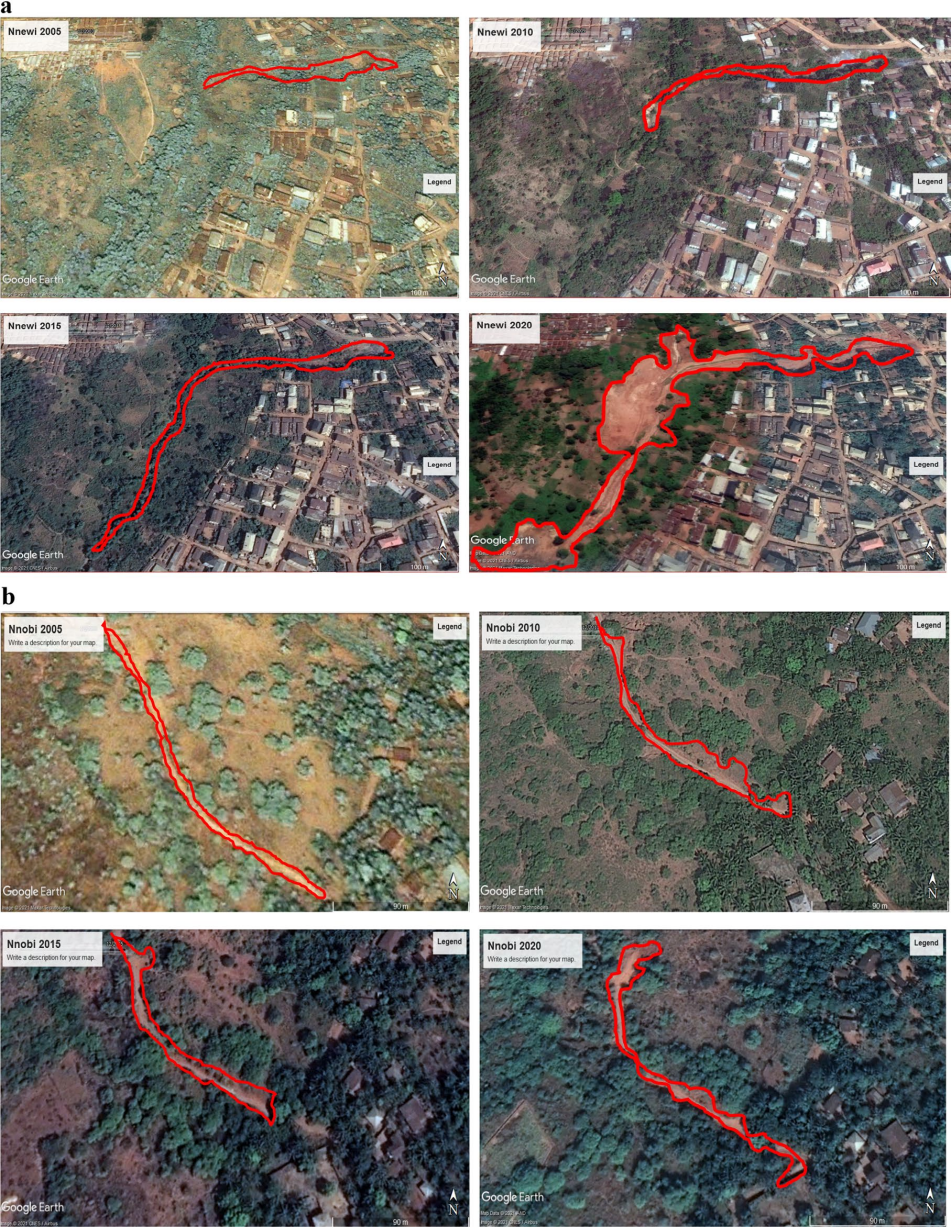

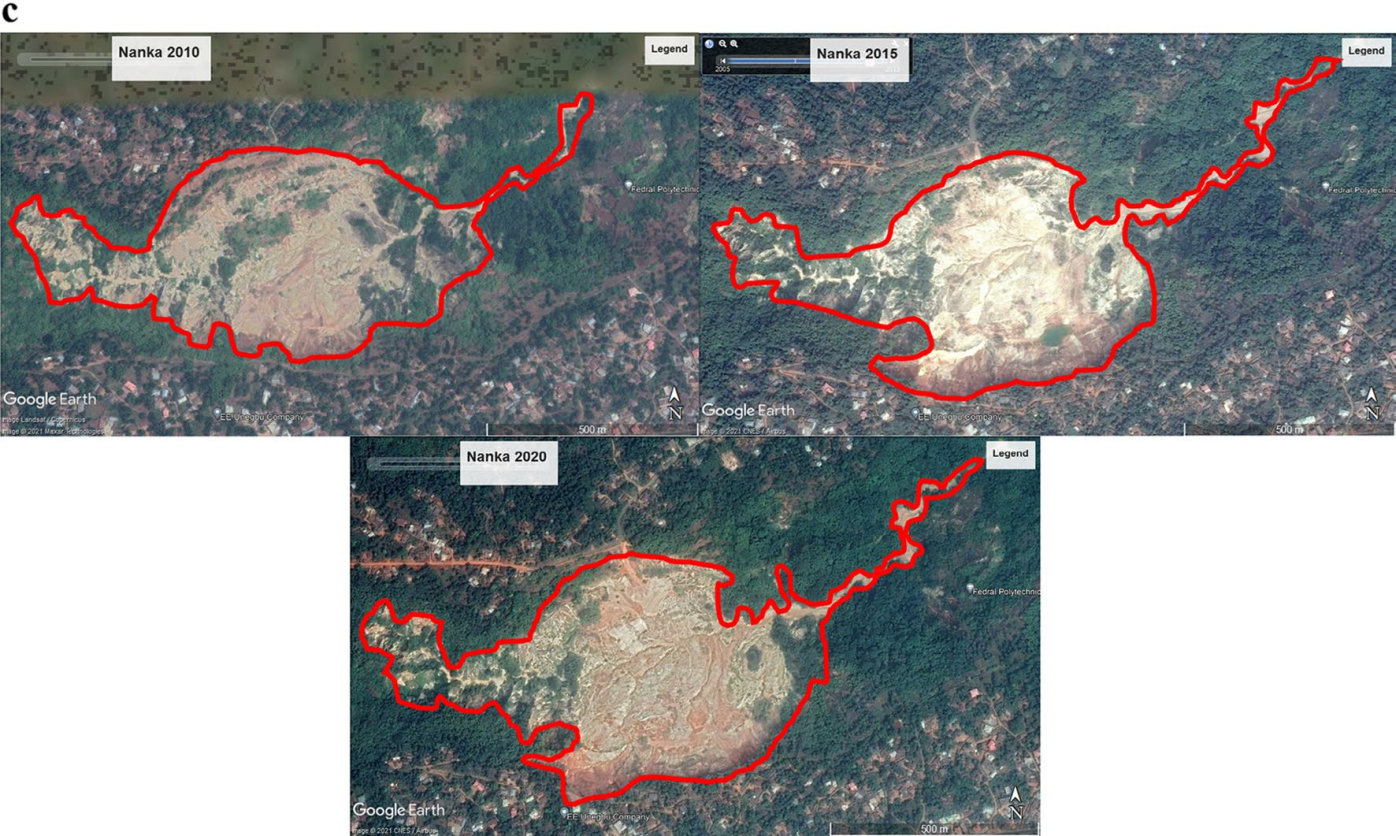
(**a**) Gully expansion rate analysis of Nnewi area from 2005 to 2020. Aerial photos obtained from the Google Earth Pro software. (**b**) Gully expansion rate analysis of Nnobi area from 2005 to 2020. Aerial photos obtained from the Google Earth Pro software. (**c**) Gully expansion rate analysis of Nanka area from 2005 to 2020. Aerial photos obtained from the Google Earth Pro software.

**Figure 13:**
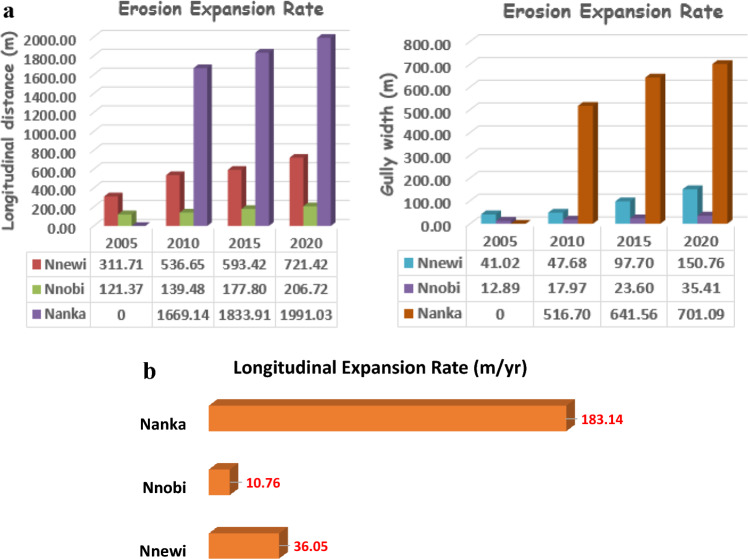
(**a**) Gully expansion rate plots. (**b**) Combined plot of the gully expansion rate (m/yr).

**Figure 14:**
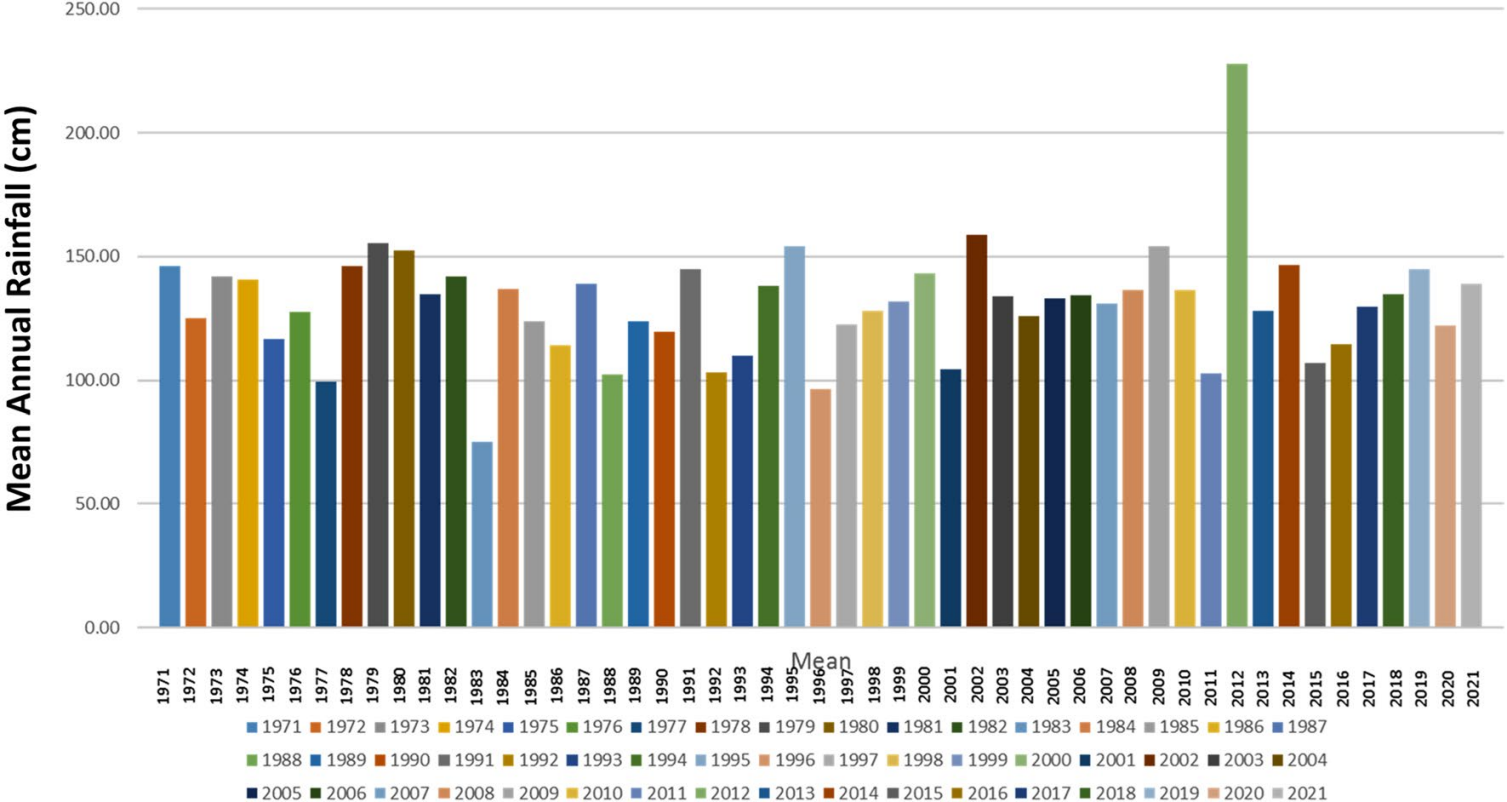
Rainfall data of the study area from 1971 to 2021.

The expansion rate of gullies was determined by measuring dimensions from Google Earth for three representative gullies: one each from Nnewi, Nnobi, and Nanka. This comparison aimed to assess the expansion rate in these locations relative to a well-known gully in the Nanka area. The findings, spanning a fifteen-year period (2005–2020), are outlined in Table 11 and depicted in Figs. 12a–c, 13a,b. The analysis disclosed that the average longitudinal expansion of the gullies was as follows: 540.80 m for Nnewi, 161.34 m for Nnobi, and 1831.36 m for Nanka. Additionally, by the end of 2020, their maximum widths were 150.76 m, 35.41 m, and 701.09 m, respectively. Evaluating the expansion rates over time, it was found that the average rates were 36.05 m/yr for Nnewi, 10.76 m/yr for Nnobi, and 183 m/yr for Nanka. This indicated that Nanka experienced the most rapid gully expansion, followed by Nnewi, and finally Nnobi. A closer examination of the rainfall data for the study area spanning January 1971 to October 2021 (Fig. 14) unveiled a gradual rise in the mean annual rainfall between 2015 and 2019, signifying an incremental accumulation of pore pressure during those years. Notably, the severe damage to buildings and the collapse of the 100 Foot road near the Okada spare parts market in Okpuno-Egbu, Nnewi (specifically location NNE 10), occurred after a heavy downpour in November 2019. In line with Arora, it’s established that higher pore pressure within geologic materials leads to reduced shear strength and bearing capacity^23^. The intense downpour likely prompted a surge in pore pressure, causing liquefaction of the loose sands in the vicinity. During liquefaction, the soil’s bearing capacity becomes nil, rendering any structure on it susceptible to failure due to shear stresses.

**Table 11:** Gully expansion rate analysis result.

Gully Location	Year	Max Width (m)	Min Width (m)	Length (m)	Ave. Longitudinal Expansion Rate (m/yr)
Nnewi	2005	41.02	19.65	311.71	**36**.**05**
	2010	47.68	21.86	536.65	
	2015	97.70	29.36	593.42	
	2020	150.76	43.41	721.42	
	Mean			**540**.**80**	
Nnobi	2005	12.89	4.43	121.37	**10**.**76**
	2010	17.97	5.31	139.48	
	2015	23.60	6.30	177.80	
	2020	35.41	7.47	206.72	
	Mean			**161**.**34**	
Nanka	2005	NB	NB	NB	**183**.**14**
	2010	516.70	40.64	1669.14	
	2015	641.56	46.85	1833.91	
	2020	701.09	50.49	1991.03	
	Mean			**1831**.**36**	

Significant values are in [bold].

### Calculation of soil erodibility factor (K)

The soil erodibility factor (K) serves as a measure of a soil’s inherent susceptibility to erosion, as outlined by Jain and Singh^24^. This factor holds significance within the framework of the Revised Universal Soil Loss Equation (RUSLE) and, importantly, it exhibits variability across seasons. Empirical observations demonstrate that K is not a constant value but instead fluctuates, with its peak during the rainy season compared to the dry season^24^. The factor K is closely linked to a soil’s physical and chemical attributes, influencing the ease with which soil particles can be dislodged. This interplay is influenced by parameters such as soil texture, aggregate stability, and permeability, which dictates the soil’s water absorption capacity^24,31^.

The soil erodibility factor (K) is given by the following equation:

$$ \begin{aligned} & {\text{K}}_{{{\text{fact}}}} = 1.292 \, [2.1 \times 10^{ - 6} {\text{F}}_{{\text{p}}}^{1.14} \left( {12 - {\text{P}}_{{{\text{om}}}} } \right) \, + \, 0.0325 \, \left( {{\text{S}}_{{{\text{struc}}}} - 2} \right) \, + \, 0.025 \, \left( {{\text{F}}_{{{\text{perm}}}} - 3} \right)] \\ & {\text{F}}_{{\text{p}}} = {\text{P}}_{{{\text{silt}}}} \left( {100 - {\text{P}}_{{{\text{clay}}}} } \right) \\ \end{aligned} $$

where F_p_ = particle size parameter. P_om_ = percent organic matter. S_struc_ = soil structure index. F_perm_ = profile-permeability class factor. P_clay_ = percent clay.

- The values for soil structure were assigned based on the soil type as follows

1 = very fine granular soil.

2 = fine granular soil.

3 = medium or coarse granular soil.

4 = blocky, platy or massive soil.

- The values for soil permeability class factor were assigned based on soil type as follows:

1 = very slow infiltration.

2 = slow infiltration.

3 = slow to moderate infiltration.

4 = moderate infiltration.

5 = moderate to rapid infiltration.

6 = rapid infiltration.

*Calculation for Nnewi*: For the Nnewi area, the following values were used:

Particle size parameter (F_p_) = Calculated based on P_silt_ and P_clay_.

Organic matter (P_om_) = 0.1

Soil structure index (S_struc_) = 2.5

Permeability class factor (F_perm_) = 5

*Calculation for Nnobi*: For the Nnobi area, the following values were employed:

Particle size parameter (F_p_) = Calculated based on P_silt_ and P_clay_.

Organic matter (P_om_) = 0.1

Soil structure index (S_struc_) = 2

Permeability class factor (F_perm_) = 3

The soil erodibility factor (K) of Nnewi area is given as:

$$ \begin{aligned} {\text{K}}_{{{\text{fact}}}} & = \, 1.292[2.1 \times 10^{ - 6} {\text{F}}_{{\text{p}}}^{1.14} \left( {12 - {\text{P}}_{{{\text{om}}}} } \right) \, + \, 0.0325 \, \left( {{\text{S}}_{{{\text{struc}}}} - 2} \right) \, + \, 0.025 \, \left( {{\text{F}}_{{{\text{perm}}}} - 3} \right)] \\ & = 1.292[2.1 \times 10^{ - 6} 3.06^{1.14} \left( {12 - 0.1} \right) \, + \, 0.0325 \, \left( {2.5 - 2} \right) \, + \, 0.025 \, \left( {5 - 3} \right)] \\ & = 8.57 \times 10^{ - 2} \\ \end{aligned} $$

The soil erodibility factor (K) of Nnobi area is given as:

$$ \begin{aligned} {\text{K}}_{{{\text{fact}}}} & = \, 1.292[2.1 \times 10^{ - 6} {\text{F}}_{{\text{p}}}^{1.14} \left( {12 - {\text{P}}_{{{\text{om}}}} } \right) \, + \, 0.0325 \, \left( {{\text{S}}_{{{\text{struc}}}} - 2} \right) \, + \, 0.025 \, \left( {{\text{F}}_{{{\text{perm}}}} - 3} \right)] \\ & = 1.292[2.1 \times 10^{ - 6} 4.12^{1.14} \left( {12 - 0.1} \right) \, + \, 0.0325 \, \left( {2 - 2} \right) \, + \, 0.025 \, \left( {3 \, - 3} \right)] \\ & = 1.62 \times 10^{ - 4} \\ \end{aligned} $$

The derived values indicated that the soils in Nnewi exhibited a notably higher erodibility potential compared to those in Nnobi.

## Conclusion

This research work has been focused on a comprehensive analysis of selected gullies situated in the regions of Nnewi and Nnobi, south-eastern Nigeria. The central aim of this study was to delve into the expansion rate and soil erodibility factor (K) of these specific gullies, shedding light on critical geotechnical attributes that influence both soil erodibility and gully intensity. The distinct objectives that guided this investigation encompassed the evaluation of geotechnical characteristics that exert an impact on soil erodibility and gully intensity, the computation of the expansion rate of the gullies and determination of the soil erodibility factor (K), the formulation of a model for slope failure, and the assessment of the factor of safety of the gully slope material.

In the process of achieving these objectives, a thorough examination of the geotechnical properties inherent to the soils within the study area was conducted. It became evident that Nnewi, in contrast to Nnobi, is positioned on a relatively steep topography. The outcome of index and geotechnical analysis revealed noteworthy distinctions between the two regions. Specifically, Nnewi exhibited lower fine content, higher sand content, greater permeability, decreased shear strength, inadequate soil compaction, and sparse vegetation coverage when compared to Nnobi. These findings collectively render the soils in Nnewi significantly more susceptible to erosive forces generated by running water, thus explaining the escalated gully erosion observed in this area.

An essential parameter, the soil erodibility factor (K), emerged as a crucial indicator of erosion susceptibility. Notably, the calculated K values for Nnewi and Nnobi were 8.57 × 10^–2^ and 1.62 × 10^–4^, respectively. This disparity in K values aligns with the disparities identified in the geotechnical property analysis, thereby reinforcing the notion that Nnewi’s soils possess a higher potential for erosion compared to Nnobi.

Assessing the gully expansion rates over a span of fifteen years (2005–2020) disclosed distinctive rates for various gully types. Specifically, Nnewi, Nnobi, and Nanka type gullies exhibited average longitudinal expansion rates of 36.05 m/yr, 10.76 m/yr, and 183.14 m/yr, respectively. This analysis underscored the role of vegetative cover, with Nnobi’s expansive vegetation contributing to its reduced susceptibility to erosive forces.

Slope stability simulations were performed to ascertain the stability of slopes across the study area. The computed factor of safety (FoS) values unveiled varying degrees of stability. For saturated conditions, FoS values ranged from 0.50 to 0.76 and 0.82 to 0.95 for Nnewi and Nnobi, respectively. Meanwhile, under unsaturated conditions, FoS values ranged from 0.73 to 0.98 for Nnewi and from 0.87 to 1.04 for Nnobi. These findings illuminated the heightened hazard levels of slopes during the rainy season when the gully slope materials experienced saturation.

The outcomes of this study contribute to a more comprehensive understanding of the dynamics of gully erosion in these regions and provide valuable insights for future erosion mitigation efforts.

## Data Availability

The datasets produced in this study are not publicly accessible as a result of a confidentiality agreement among the authors. This agreement was implemented to safeguard the data from any unauthorized usage or publication. Nevertheless, the corresponding author will be pleased to provide the data upon reasonable request. Interested parties are encouraged to reach out directly to the corresponding author for further information on accessing the datasets.
